# Two mouse models of Alzheimer’s disease accumulate amyloid at different rates and have distinct Aβ oligomer profiles unaltered by ablation of cellular prion protein

**DOI:** 10.1371/journal.pone.0294465

**Published:** 2023-11-17

**Authors:** Silvia A. Purro, Michael Farmer, Elizabeth Noble, Claire J. Sarell, Megan Powell, Daniel Yip, Lauren Giggins, Leila Zakka, David X. Thomas, Mark Farrow, Andrew J. Nicoll, Dominic Walsh, John Collinge

**Affiliations:** 1 MRC Prion Unit at UCL, Institute of Prion diseases, University College London, London, United Kingdom; 2 Laboratory for Neurodegenerative Research, Ann Romney Center for Neurologic Diseases, Brigham and Women’s Hospital and Harvard Medical School, Boston, Massachusetts, United States of America; INRA Centre de Jouy-en-Josas, FRANCE

## Abstract

Oligomers formed from monomers of the amyloid β-protein (Aβ) are thought to be central to the pathogenesis of Alzheimer’s disease (AD). Unsurprisingly for a complex disease, current mouse models of AD fail to fully mimic the clinical disease in humans. Moreover, results obtained in a given mouse model are not always reproduced in a different model. Cellular prion protein (PrP^C^) is now an established receptor for Aβ oligomers. However, studies of the Aβ-PrP^C^ interaction in different mouse models have yielded contradictory results. Here we performed a longitudinal study assessing a range of biochemical and histological features in the commonly used J20 and APP-PS1 mouse models. Our analysis demonstrated that PrP^C^ ablation had no effect on amyloid accumulation or oligomer production. However, we found that APP-PS1 mice had higher levels of oligomers, that these could bind to recombinant PrP^C^, and were recognised by the OC antibody which distinguishes parallel, in register fibrils. On the other hand, J20 mice had a lower level of Aβ oligomers, which did not interact with PrP^C^ when tested *in vitro* and were OC-negative. These results suggest the two mouse models produce diverse Aβ assemblies that could interact with different targets, highlighting the necessity to characterise the conformation of the Aβ oligomers concomitantly with the toxic cascade elicited by them. Our results provide an explanation for the apparent contradictory results found in APP-PS1 mice and the J20 mouse line in regards to Aβ toxicity mediated by PrP^C^.

## Introduction

Neurofibrillary tangles composed of tau protein, and plaques of amyloid β-protein (Aβ) are pathognomonic for Alzheimer’s disease (AD). Although plaques can be toxic to nearby dendrites [1], it has been suggested that the main toxic effects are imparted by soluble aggregated forms of Aβ, referred to as oligomers. Many putative receptors for Aβ oligomers have been described [2–4]. Of these candidates, the cellular prion protein (PrP^C^) has the most supportive evidence [4]. Multiple labs additionally described two Aβ binding sites present on PrP^C^, identified signalling pathways activated by the interaction, and demonstrated that PrP^C^ ablation, or inhibition mediated by anti-PrP^C^ antibodies, prevents Aβ associated synaptotoxicity *in vitro* and *in vivo*, providing therapeutic proof of principle [5–11] such that PrP^C^ is now a recognised and validated receptor for Aβ in the AD field. While there is near consensus agreement that PrP^C^ binds Aβ oligomers preferentially and with high affinity [7, 12], there has been controversy about the requirement of PrP^C^ to mediate the synaptotoxic effects of Aβ.

Initially it was found that ablation of PrP^C^ rescued memory impairment, synaptic dysfunction and premature survival in the APP_swe_-PS1ΔE9 AD mouse model (APP-PS1) [12, 13], but shortly afterwards, it was reported that deletion of PrP^C^ in a different AD mouse model, J20 transgenic mice, did not alter any of the parameters measured, and by contrast actually accelerated premature death in these animals [14]. Both mouse models overexpress human amyloid precursor protein (APP), albeit under different promoters and harbouring different mutations. APP-PS1 mice express two different *Prnp* promoter driven transgenes expressing APP with the Swedish mutation together with presenilin 1 (PS1) with an exon 9 deletion [15, 16]; whereas J20 mice express APP with Swedish and Indiana familial AD mutations under the platelet-derived growth factor subunit β (PDGF-β) promoter [17].

Unfortunately, APP transgenic mouse models do not fully recapitulate neurodegeneration phenotypes. This has been a huge drawback in the field for years. The first generation of AD mouse models overexpress proteins such as mutated APP and/or PS1 to accelerate AD phenotypes within the lifespan of the mouse. Such models differ in several ways, including Aβ plaque burden, localisation, and timing of deposition possibly due to Aβ kinetics and level of expression, leading to differences in the onset of memory impairments, synaptic dysfunction, neuronal death, presence of tau tangles, etc. [18]. Therefore, different pathological AD pathways may be variably present and active in different AD mouse models, and the success of targeting a specific pathway may depend on the characteristics of the individual model. Here, we directly compared the APP-PS1 and J20 mouse lines, studied previously by different laboratories with conflicting results, to independently assess the impact of PrP^C^ deletion on their respective phenotypes, via immunohistochemical and biochemical analyses. We found that APP-PS1 mice produce high levels of Aβ oligomers with a conformation that binds to PrP^C^, in contrast with J20 mice, which produce lower levels of PrP^C^-binding oligomers. It is perhaps therefore unsurprising that the J20 AD phenotype is unaffected by PrP^C^ expression. This result suggests that the J20 line is not suitable for investigating the Aβ-PrP^C^ interaction. Moreover, it further highlights the diversity of Aβ oligomers and the necessity to study aggregate conformations, their respective ability to bind to cellular receptors, and their possible function and toxicity.

## Materials and methods

### Reagents

All chemicals and reagents were purchased from Sigma-Aldrich unless otherwise noted. Synthetic Aβ_1–42_ was synthesized and purified by Dr. James I. Elliott at the ERI Amyloid laboratory Oxford, CT, USA. Peptide mass and purity (>99%) were confirmed by reversed-phase HPLC and electrospray/ion trap mass spectrometry.

### Mice

Work with animals was performed under licence granted by the UK Home Office (PPL 70/9022) and conformed to University College London institutional and ARRIVE guidelines. APP_swe_-PS1ΔE9 mice (APP-PS1, JAX MMRRC Stock# 034829; [15]) were obtained from Professor Strittmatter’s laboratory, and J20 mice [17] were sourced from The Jackson Laboratory (JAX MMRRC Stock # 034836). Both lines were crossed with either C57BL/6J (Charles River, Margate, UK) or PrP^C^ null backcrossed onto a C57BL/6 background (B6.129S7-Prnp^tm1Cwe^/Orl, EMMA Stock # 01723; [19]) to generate the mouse lines required. Non-transgenic littermates from these crosses were used to populate control groups. Mice were culled at 3, 6 and 12 months old. Groups of 5–8 male mice were used for biochemical and histological analysis. Mice were anesthetized with isoflurane/O_2_ and decapitated. Brains were removed, divided by a sagittal cut with half brain frozen and the other half fixed in 10% buffered formal saline. Subsequent immunohistochemical investigations were performed blind to sample provenance. The genotype of each mouse was determined by PCR of ear punch DNA and all mice were uniquely identified by sub-cutaneous transponders. RT-PCR was used for determining J20 transgene copy number.

### Immunohistochemistry

Fixed brain was paraffin wax embedded. Serial sections of 5 μm nominal thickness were pre-treated by immersion in 98% formic acid for 8 mins followed by Tris-EDTA buffer for antigen retrieval. All sections were stained with Hematoxylin and Eosin for morphological assessment. Aβ deposition was visualized using biotinylated 82E1 (cat n.10326, IBL) as the primary antibody, using Ventana Discovery automated immunohistochemical staining machine (ROCHE Burgess Hill, UK) and proprietary solutions. Visualization was accomplished with diaminobenzidine staining.

Histological slides were digitised on a LEICA SCN400F scanner (LEICA Milton Keynes, UK) at x40 magnification and 65% image compression setting during export. Slides were archived and managed on LEICA Slidepath (LEICA Milton Keynes, UK). For the preparation of light microscopy images, image captures were taken from Slidepath. Publication figures were assembled in Adobe Photoshop.

### Digital image analysis for Aβ quantification

Digital image analysis was performed using Definiens Developer 2.3 (Munich). Initial tissue identification was performed using x10 resolution and stain detection was performed at x20 resolution.

#### Tissue detection

Initial segmentation was performed to identify all tissue within the image, separating the sample from background ‘glass’ regions for further analysis. This separation was based on a grey-scale representation of brightness composed of the lowest (darkest) pixel value from the three comprising colour layers (RGB colour model). A dynamic threshold was calculated using the 95^th^ centile which represents the threshold separating the 5% of area with the brightest/highest intensity from the darker 95%; this was then adjusted by -10 (256 colour scale) to ensure accurate tissue separation–this adjustment is necessary to prevent the inclusion of non-tissue regions that, although comprising unstained background, have a reduced pixel value.

#### Stain detection

Identification of brown staining is based on the transformation of the RGB colour model to a HSD representation [20]. This provides a raster image of the intensity of each colour of interest (Brown and Blue). Subtraction of the blue stain from the brown stain intensity at each pixel gives a third raster image, Brown+ve, with a positive number where brown stain is prevalent.

All areas with brown staining above 0.15 arbitrary units (au), and Brown+ve greater than 0.1, were identified as *Brown Area*. This *Brown Area* was then subdivided to identify *Light Brown Area* < 0.5 au < = *Dark Brown Area*.

Each *Dark Brown Area* was used as a seed for plaques, by growing them into any connected *Light Brown Area*. Plaques were removed if they did not meet several criteria: smaller than 10μm^2^; contained less than 1.5μm^2^ of *Dark Brown Area*; high stain intensity (>0.5 au) and low standard deviation (<0.25 au); area less than 40 μm^2^ with a non-elliptical shape; or area greater than 40 μm^2^ with greater than 70% *dark brown area*.

#### Tissue selection

Brain regions were manually selected by hand and plaque and stain coverage data exported per region.

### Aβ preparations (ADDLs)

Aβ-derived diffusible ligands (ADDLs) were prepared as described previously [10]. Briefly, 20–25 mg of dry weight peptide was dissolved in 2% w/v anhydrous DMSO for 5 minutes and then diluted to 0.5 mg/ml in phenol red-free Ham’s F12 medium without L-glutamine (Caisson Labs), vortexed for 15 seconds and incubated at room temperature overnight without shaking. After 24–36 h aliquots were tested for the presence of large protofibrillar aggregates using size-exclusion chromatography (GE Healthcare). Fractions containing less than 20% monomer, were centrifuged at 16000 *g* for 20 minutes at 4°C and the upper 90% of the supernatant collected, snap frozen in liquid nitrogen and stored at -80°C in aliquots.

### Brain homogenates

Brain samples were homogenised using a cell homogeniser PreCellys24 (Bertin) and whole brain homogenates were prepared at 10% weight in volume (w/v) in PBS with protease and phosphatase inhibitors (Pierce). Homogenates were clarified by centrifugation for 5 minutes at 1000 x*g*. Benzonase treatment was performed as per below when required. Bradford quantification of total protein was carried out to ensure similar amounts of proteins were used on the biochemical assays.

### Western blotting

Brain homogenate was thawed on ice for 10 minutes, diluted to a final concentration of 2 mg/ml total protein in PBS as measured by Bradford assay, and added to 2x SDS sample buffer. Samples were boiled for 5 minutes then electrophoresed in pre-cast 4–12% NuPAGE Bis-Tris Gels (Invitrogen). Following transfer, nitrocellulose membranes (Amersham GE Lifesciences) were incubated in Licor Odyssey blocking buffer (#927–40000) for 1 h at RT. Membranes were washed 3 x 10 minutes in PBST (PBS, 0.05% (v/v) Tween-20), then incubated overnight at 4°C in primary antibodies. APP was labelled using 22C11 Merck Millipore #MAB348 (1:5000), GAPDH was labelled using Sigma G945 (1:50,000) and PrP^C^ labelled using ICSM18 D-Gen (final concentration 3 μg/ml). After incubation in primary antibody, membranes were washed 3 x 10 minutes in PBST, then incubated in secondary antibodies (Odyssey Goat anti-mouse IRDye 800CW or anti-rabbit 680LT) for 1 h at RT. Membranes were then washed twice in PBST, once in PBS and immunoreactive bands were detected and quantified using a Licor Odyssey imaging system (Licor Biosciences).

### Immunoassay to detect PrP^C^ binding Aβ species

To detect PrP^C^ binding Aβ species a plate-based DELFIA (Dissociation-enhanced lanthanide fluorescent immunoassay) was used. HuPrP 23–111 was expressed and purified as described previously for HuPrP 23–231 [21]. Briefly, inclusion bodies were re-suspended in 6M GdnHCl, β-mercaptoethanol, loaded onto a NiNTA column and refolded by stepwise oxidation. Following elution and dialysis the His tag was cleaved using thrombin, the protein loaded again onto a NiNTA column and eluted in 20mM Bis-Tris, 600mM Imidazole, pH6.5. After dialysis in 20mM Bis-Tris, pH6.5 the protein was stored in aliquots at -80°C.

Thirty microliters of 1 μM human PrP23–111 (10 mM sodium carbonate, pH 9.6) was bound to high binding 384-well white plates (Greiner #G781074) with shaking at 400 RPM for 1 h at 37°C, washed with 3 x 100 μl of PBST (0.05% Tween-20), blocked with 100 μl Superblock (Thermo Scientific) with shaking at 400 RPM at 37°C for 1 h and washed with 3 x 100 μl of PBST. Synthetic ADDL preparations were used as standards. Thirty microlitres of benzonase treated 10% brain homogenates (all normalised to the sample with the lowest concentration of protein) were incubated for 1 h at 25°C with shaking at 400 RPM and washed with 3 x 100 μl of PBST. Aβ oligomers were detected by 30 μl of 0.2 mg/ml 82E1 in DELFIA assay buffer (PerkinElmer) for 1 h at 25°C with shaking at 400 RPM, washed with 3 x 100 μl of PBST, then incubated for 30 min at 25°C with shaking at 400 RPM with 9 ng/well of DELFIA Eu-N1 anti-mouse antibody in DELFIA assay buffer (PerkinElmer), washed with 3 x 100 μl of PBST before enhancing with 80 μl of DELFIA Enhancement Solution (PerkinElmer). Plates were scanned for time-resolved fluorescence intensity of the europium probe (λex 320 nm, λem 615 nm) using a PerkinElmer EnVision plate reader.

### Dot blot analysis

One microliter of mouse brain homogenate (2 μg total protein) or synthetic ADDL preparation (5 ng) was spotted directly onto dry nitrocellulose membrane (Amersham) and air dried for 1 h before blocking overnight at 4°C in 5% (w/v) non-fat dried milk in PBST (PBS, 0.1% (v/v) Tween-20). Following three 10 min washes with PBST, membranes were incubated with OC antibody (Millipore, #AB2286) (1:4000 dilution) diluted in 5% (w/v) non-fat dried milk in PBST overnight at 4°C. Following three 10 min washes with PBST, membranes were incubated with IRDye 800CW donkey anti-rabbit IgG antibody in Odyssey Blocking buffer (Licor) for 1 h at RT. The membrane was then visualised using an Odyssey scanner. Membranes were subsequently stripped (Restore Plus, Invitrogen) and re-blotted for the loading control β-Actin.

### Multiplex Aβ peptide immunoassay

Levels of Aβ peptides in the mouse brain homogenates were determined using a Multiplex Aβ peptide panel (6E10) immunoassay (Meso Scale Discovery (MSD), Rockville, MD), according to the manufacturer’s instructions. Before analysis, homogenates were incubated with Guanidine-HCl (6M final concentration) to disaggregate preformed Aβ aggregates for 1 hour. All incubations were carried out at room temperature on a plate shaker at 600 RPM. All standards and samples were diluted in PBS and loaded in duplicate. Aβ peptide levels were determined using the MESO QUICKPLEX SQ 120 and analysed using the MSD Workbench 4.0 software.

### Oligomeric Aβ immunoassay

1C22 is an oligomer-preferring antibody [22], that was used previously to detect Aβ oligomers in mouse and human brain [23, 24]. MULTI-ARRAY® 96 well standard bind microplates (MSD) were coated with the monoclonal antibody 1C22 (2 μg/ml) diluted in PBS and incubated at 4°C overnight. Wells were blocked with 5% (w/v) Blocker A (MSD) for 1h. Synthetic ADDL preparations were used as standards to generate a twelve-point standard curve. All samples and standards were diluted in PBS and loaded in duplicate (25 μl/well). Biotinylated 82E1 (2 μg/ml) diluted in assay diluent (1% Blocker A/PBST) was used for detection. Bound biotinylated 82E1 was measured using SULFO-TAG streptavidin (MSD) diluted in assay diluent. Light emitted from the SULFO-TAG at the electrode surface was detected using the MESO QUICKPLEX SQ 120 imager. All incubations were performed at room temperature on a plate shaker at 600 RPM and all wash steps between incubations were performed using 150 ul PBST, unless stated otherwise. Data were analysed using the MSD Workbench 4.0 software. The limit of detection (LOD) is defined as: LOD = 2.5 x standard deviation of the background. The lower limit of reliable quantification (LLOQ) is defined as the lowest standard with a percentage back interpolation of 100 ± 20%, a percentage coefficient of variance (CV) ≤ 20% and a mean blank signal higher than the mean blank signal + (9 * standard deviations of the blank signal). The average of three independent experiments: LLOD 14.1 ± 8.3 (pg/ml) and LLOQ 101.7 ± 55.5 (pg/ml).

### Statistical analysis

All statistical analysis and graphs were generated using the statistical package GraphPad PRISM v7 (GraphPad Software, Inc., La Jolla, USA). For multiple comparisons, graphs depict median values and Kruskal-Wallis was used and corrected for multiple comparisons using Dunn’s multiple comparison test. Statistical significance was set to P < 0.05.

## Results

### Aβ aggregation is independent of PrP^C^ expression

**Fig 1:**
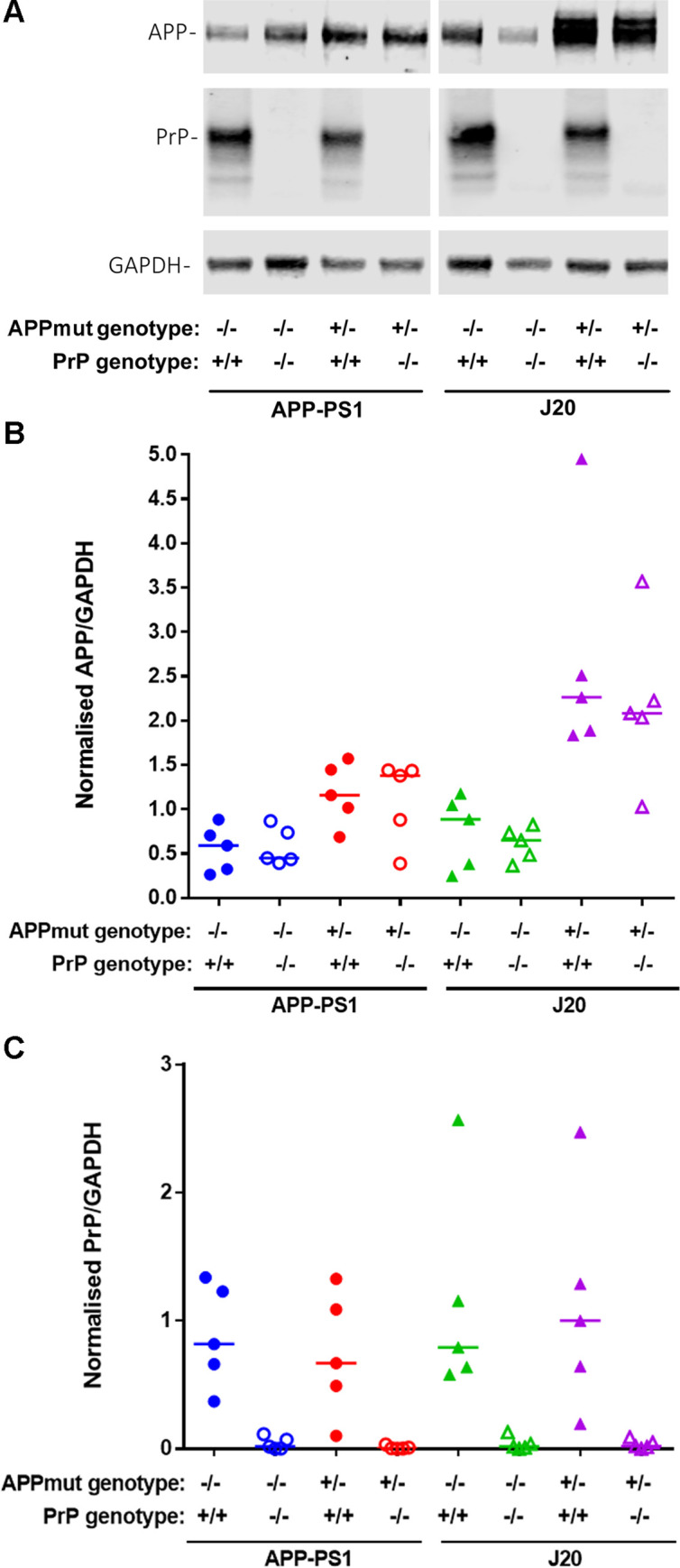
Knockout of PrP^C^ does not affect total APP levels in wild type or AD-model mice. **A)** Total brain homogenate from 12-month old mice across 8 genotypes were analysed via western blot. APP was labelled using the N-terminal monoclonal antibody 22C11, and PrP^C^ labelled using ICSM18. n = 5. **B)** Quantification of APP expression levels determined by western blot. J20 mice showed significantly higher total APP expression than APP-PS1 mice irrespective of PrP^C^ status (APP-PS1 vs J20, p = 0.024), however no significant differences were observed between the PrP^C^ +/+ and -/- variants of any APP genotype. n = 5. **C)** Quantification of PrP^C^ expression levels determined by western blot. No significant differences in PrP^C^ expression were observed between APP genotypes. Deletion of PrP^C^ resulted in a significant difference on PrP^C^ expression levels for all the lines used in this study, when compared to their respective controls (WT vs PrP^C^ KO, p = 0.036; APP-PS1 vs APP-PS1 PrP^C^ KO, p = 0.019; WT vs PrP^C^ KO, p = 0.019; J20 vs J20 PrP^C^ KO, p = 0.026). n = 5.

APP-PS1 mice overexpress APP encoding the Swedish mutation plus PS1 with deletion of exon 9 [15, 16]. In contrast, J20 mice overexpress only APP with Swedish and Indiana mutations [17]. In order to assess the role of PrP^C^ in both AD mouse models, we crossed the APP-PS1 and J20 mice with a PrP^C^ knock-out (KO) mouse line. Wild-type or PrP^C^ KO littermates generated without expressing APP or PS1 transgenes were used as controls (Fig 1A). PrP^C^ expression did not alter the expression of APP, nor did the overexpression of the mutated genes APP or APP/PS1 induce any changes in PrP^C^ levels (Fig 1B and 1C).

For the four mouse lines (APP-PS1, J20, APP-PS1 PrP^C^ KO and J20 PrP^C^ KO) and their respective control littermates (WT and PrP^C^ KO) we collected brain samples at 3, 6 and 12 months of age and, then examined Aβ species and aggregation using immunohistochemical and biochemical techniques.

**Fig 2:**
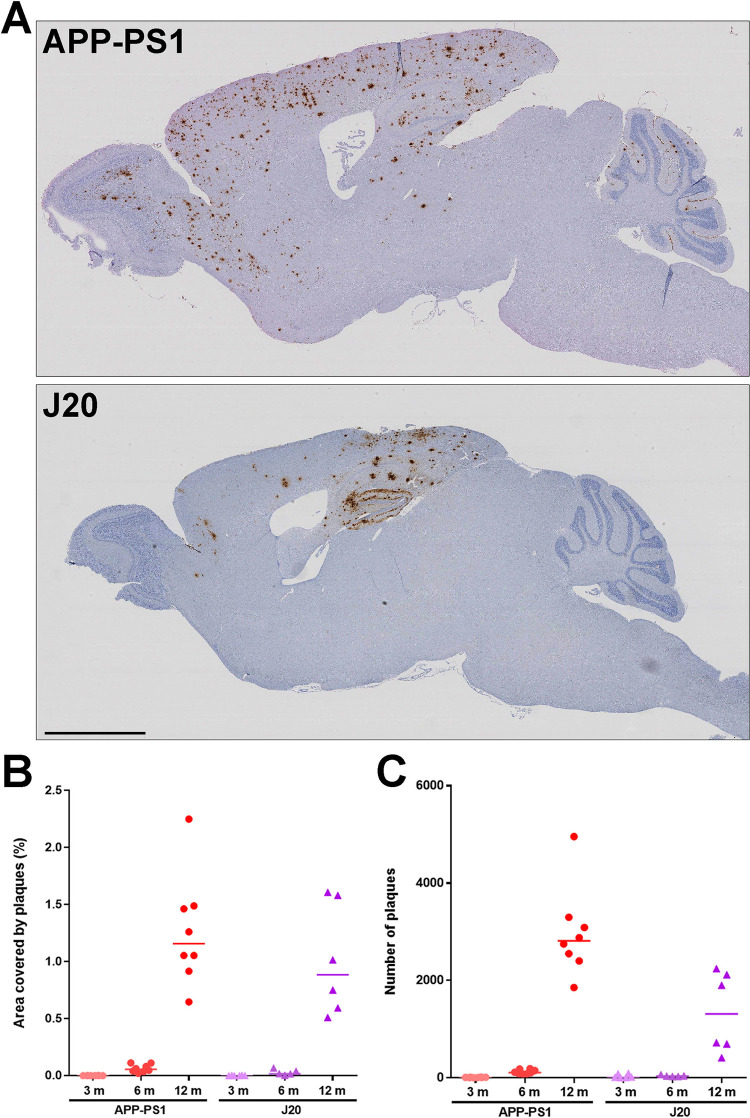
Progressive brain deposition of Aβ plaques in APP-PS1 and J20 mice. **A)** Representative images of APP-PS1 and J20 12-month old mice stained with 82E1b anti-Aβ antibody. Scale bar: 2 mm. **B)** Quantification of Aβ plaques area during aging. n = 5–8. **C)** Quantification of Aβ plaques number. APP-PS1 mice exhibited a higher number of plaques at 12 months of age (APP-PS1 vs J20, p = 0.014). n = 5–8.

**Fig 3:**
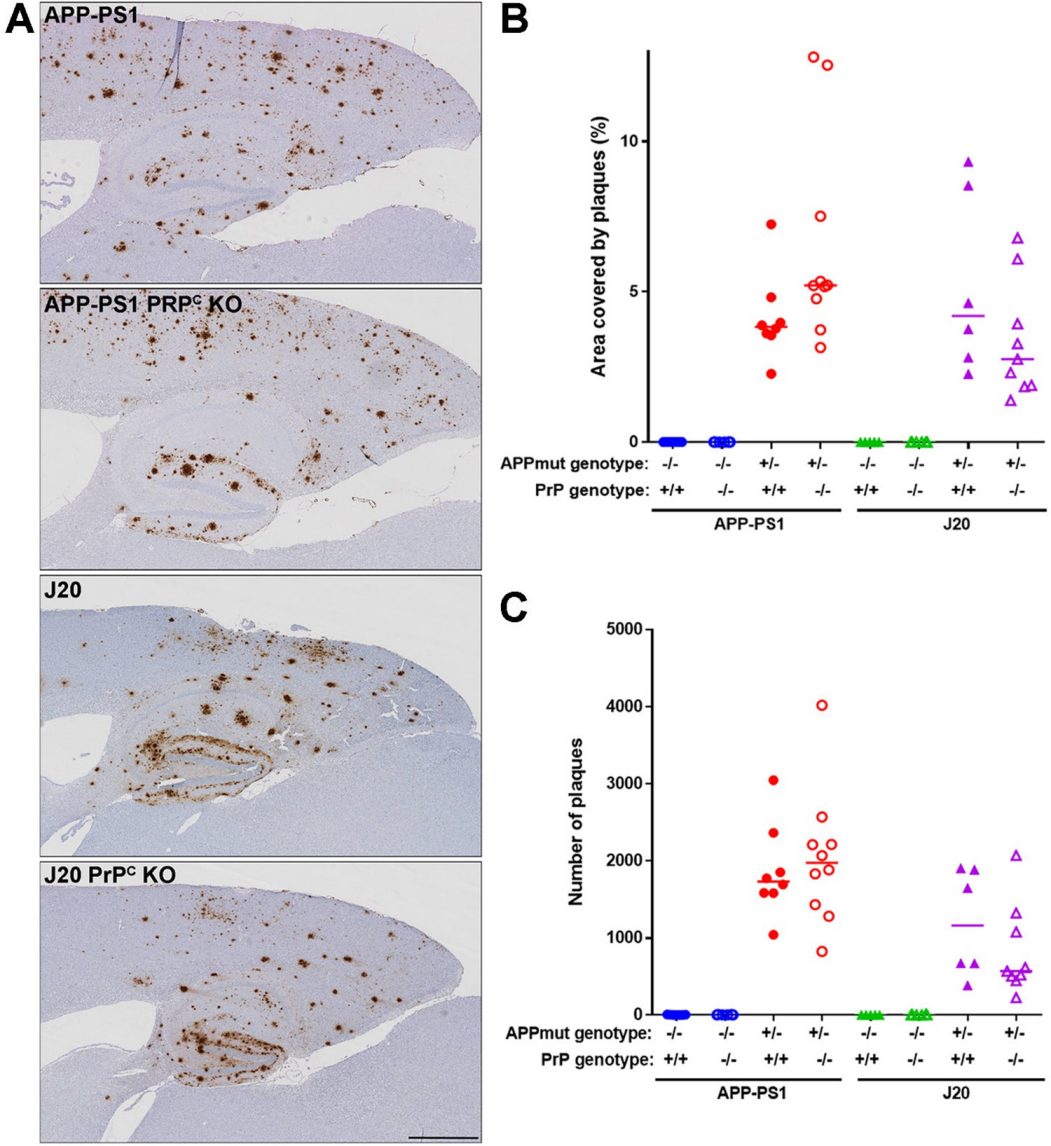
Deposition of Aβ plaques in APP-PS1 and J20 mice is independent of PrP^C^ expression. **A)** Representative images showing hippocampus, cortex and corpus callosum at 12 months of age of the four mouse lines studied. Scale bar: 700 μm. **B)** Quantification of the area covered by plaques on the above mentioned areas. For all the APP mutant lines there was a significant difference in area covered when compared to their respective controls (WT vs APP-PS1, p = 0.02; PrP^C^ KO vs APP-PS1 PrP^C^ KO, p = 0.0005; WT vs J20, p = 0.0006; PrP^C^ KO vs J20 PrP^C^ KO, p = 0.037) and, similar amounts when compared APP mutant lines to their respective ablated PrP^C^ line (APP-PS1vs APP-PS1 PrP^C^ KO, p = 0.75; J20 vs J20 PrP^C^ KO, p>0.99). n = 5–9 **C)** Quantification of the number of plaques on the above mentioned areas. Transgenic mice exhibited a higher number of plaques compared to their respective wild-type littermates (WT vs APP-PS1, p = 0.005; PrP^C^ KO vs APP-PS1 PrP^C^ KO, p = 0.002; WT vs J20, p = 0.0008; PrP^C^ KO vs J20 PrP^C^ KO, p = 0.031) and no significant difference when compared to their respective ablated PrP^C^ line (APP-PS1vs APP-PS1 PrP^C^ KO, p>0.99; J20 vs J20 PrP^C^ KO, p>0.99). n = 5–9.

Deposition of Aβ in the brains of J20 mice is visible after 6 months of age with plaques concentrated in the hippocampus, corpus callosum and cortex. Minimal plaques appear in the cerebellum even after 12 months of age. By contrast, plaques in APP-PS1 mice are more widely spread over the brain, being readily detected in cerebral cortex, olfactory bulb, hippocampus, corpus callosum and cerebellum. APP-PS1 whole brain sagittal sections exhibit twice as many plaques at 12 months than the J20 mice (APP-PS1 median: 2815 plaques, J20 median: 1315 plaques, p = 0.014) whilst brain area plaque coverage is comparable between lines (APP-PS1 median: 1.16%, J20 median: 0.88%, p = 0.75) (Fig 2A–2C). Ablation of PrP^C^ did not alter the number, location or area covered by plaques in either AD mouse model (Fig 3A–3C), in agreement with previously published results in APP-PS1 mice [13].

**Fig 4:**
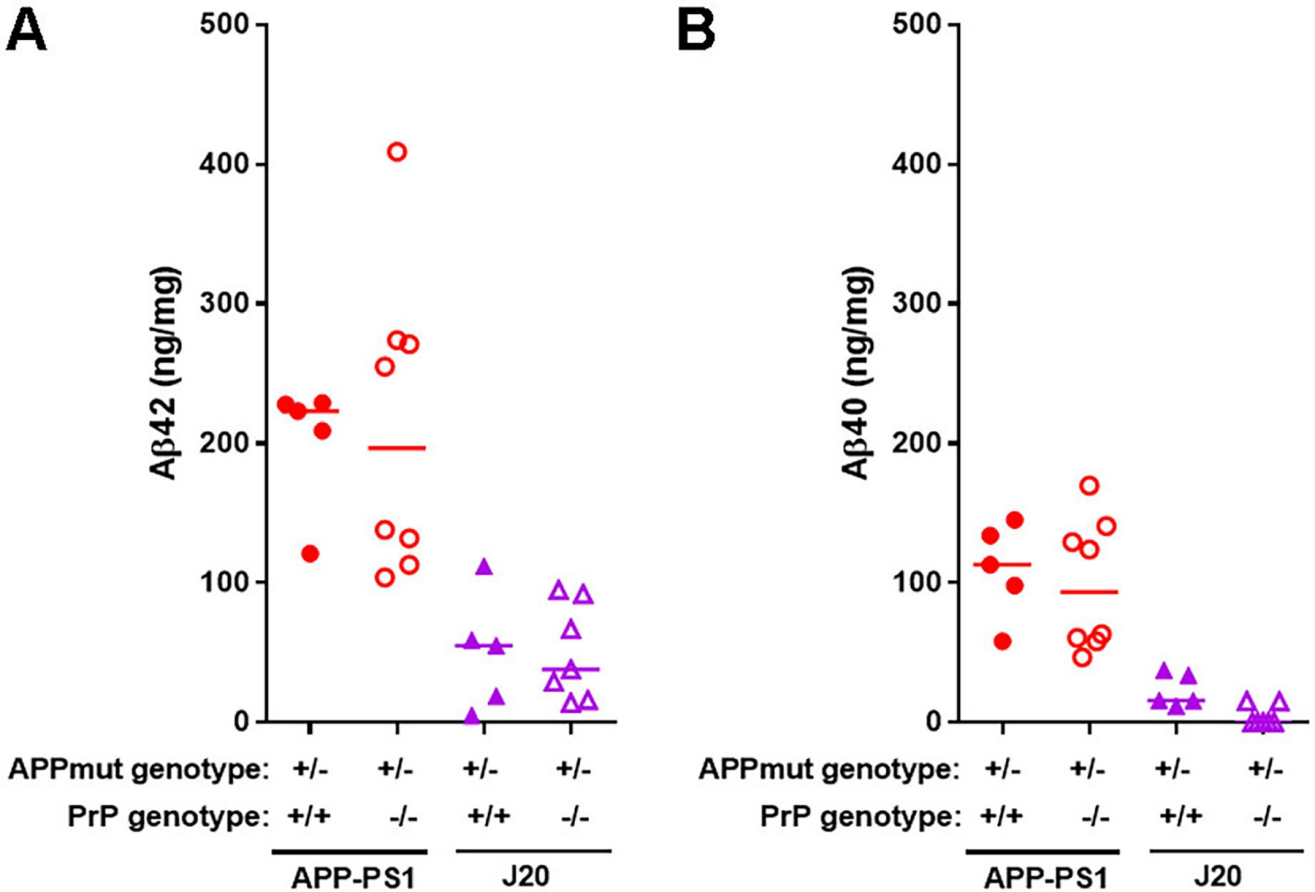
Immunoassays demonstrate that total Aβ peptide levels in APP-PS1 and J20 mouse brain are independent of PrP^C^ expression. Total brain homogenates from mice at 12 months of age were analysed by multiplex Aβ peptide panel (6E10) immunoassay from MSD. **A)** APP-PS1 expressing or not PrP^C^ presented higher levels of Aβ_42_ than J20 samples (APP-PS1 vs J20, p = 0.04; APP-PS1 PrP^C^ KO vs J20 PrP^C^ KO, p = 0.004). n = 5–8. **B)** Quantification of Aβ_4o_ levels revealed no changes due to ablation of PrP^C^ in any of the mouse lines. Levels of Aβ_4o_ were not significantly different between APP-PS1 and J20 mice (APP-PS1 vs J20, p = 0.14). n = 5–8.

Total Aβ peptides in whole brain homogenates collected at 6 and 12 months were quantified by immunoassay. At 12 months old, APP-PS1 mice had significantly more Aβ_42_ than J20 mice (for Aβ_42_ APP-PS1 median: 223 ng/mg, J20 median: 55 ng/mg, p = 0.04), but did not reach significance for the Aβ_40_ peptide (for Aβ_40_ APP-PS1 median: 113 ng/mg, J20 median: 16 ng/mg, p = 0.14); with PrP^C^ having no impact on Aβ peptide levels in either model (Fig 4). The amount of Aβ_40_ and Aβ_42_ peptides in APP-PS1 mice was almost 90% lower at 6 months versus 12 months of age (Aβ_42_ APP-PS1 median: 19 ng/mg, Aβ_40_ APP-PS1 median: 11 ng/mg, at 6 months).

**Fig 5:**
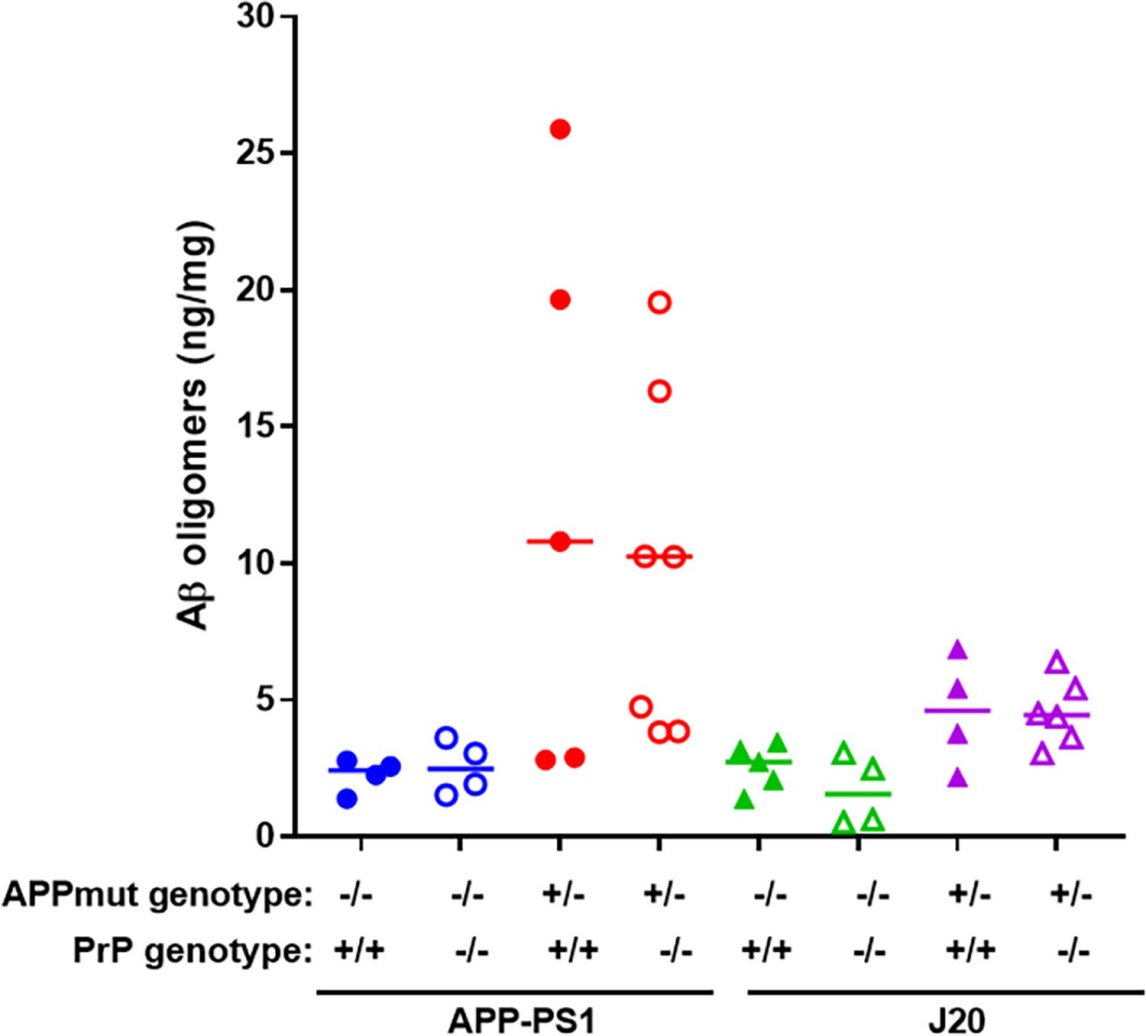
1C22-detected Aβ oligomers levels in APP-PS1 and J20 mouse brain are not altered by PrP^C^ ablation. Total brain homogenates were assayed using 1C22 anti-Aβ oligomer antibody (WT vs APP-PS1, p = 0.035; APP-PS1 vs APP-PS1 PrP^C^ KO, p>0.99; WT vs J20, p = 0.25; J20 vs J20 PrP^C^ KO, p>0.99). n = 4–7.

**Fig 6:**
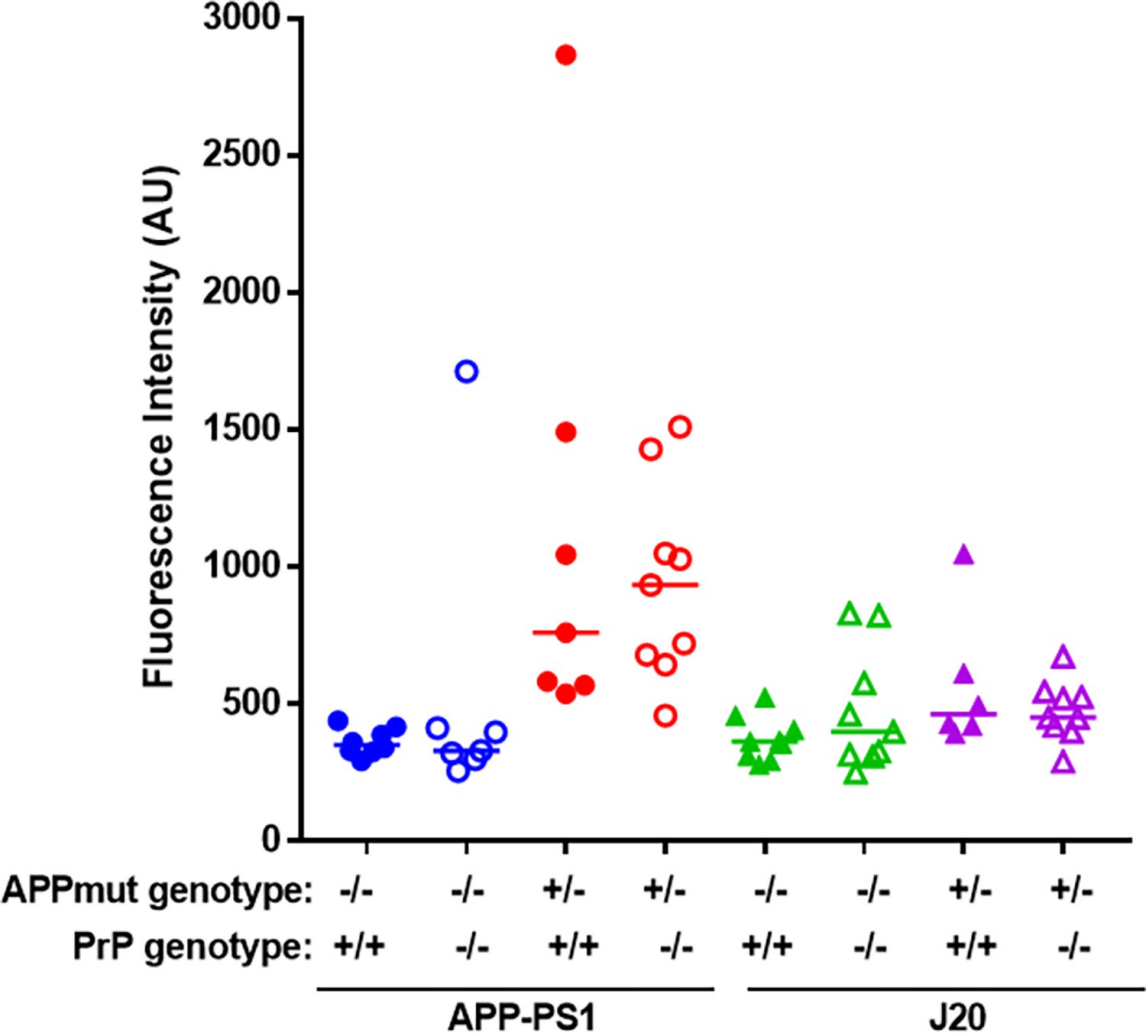
Aβ oligomers that bind to PrP^C^ are present in APP-PS1 at higher levels than in J20 mouse brain, but do not change due PrP^C^ expression. Total brain homogenates were analysed by DELFIA immunoassay to detect PrP^C^-binding Aβ species (APP-PS1 vs J20, p = 0.037; WT vs APP-PS1, p = 0.009; APP-PS1 vs APP-PS1 PrP^C^ KO, p>0.99; WT vs J20, p = 0.12; J20 vs J20 PrP^C^ KO, p>0.99). n = 6–9.

**Fig 7:**
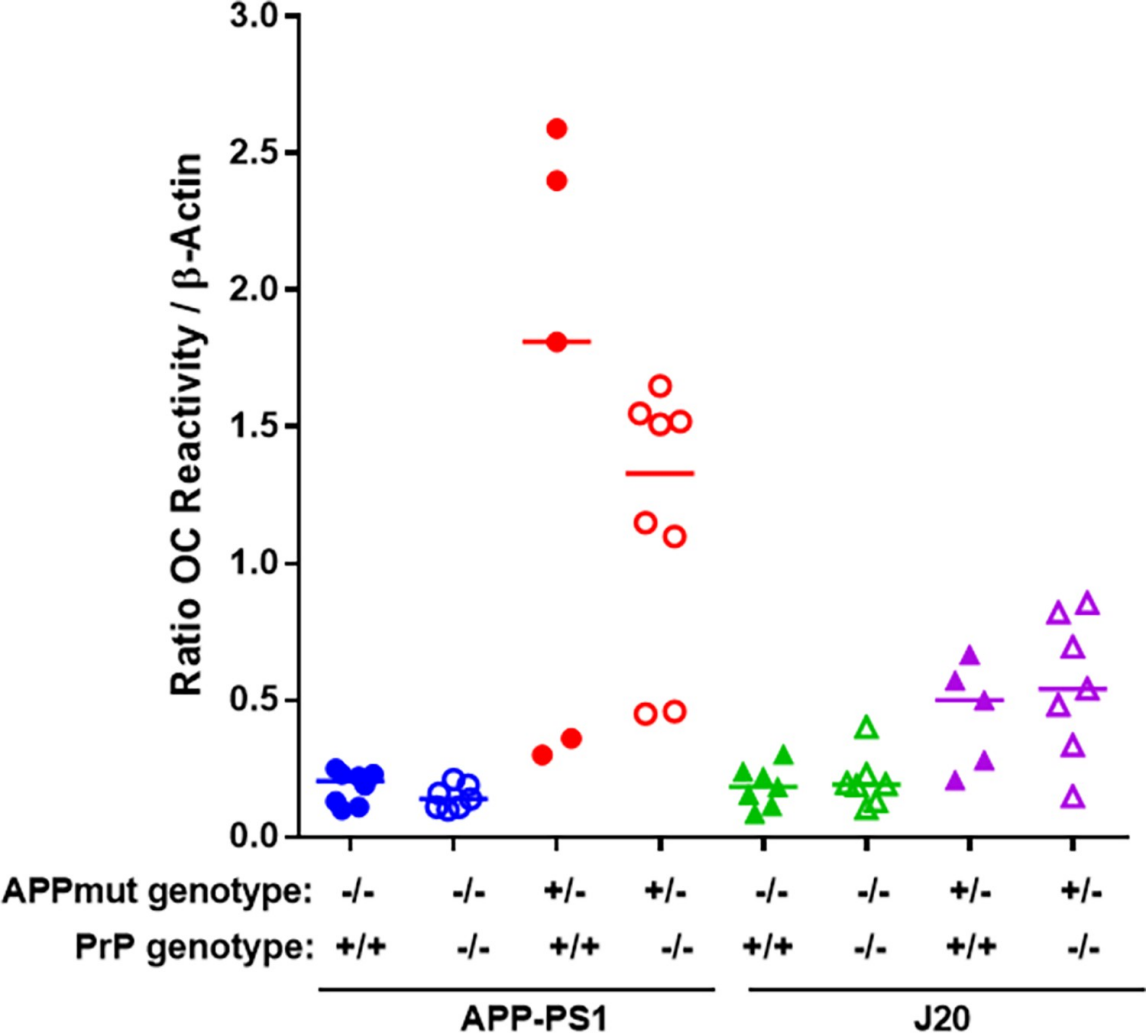
Aβ oligomers present in APP-PS1 and J20 mouse brain have different conformations, independent of PrP^C^ expression. Total brain homogenates were quantified by dot blot using OC antibody (WT vs APP-PS1, p = 0.012; APP-PS1 vs APP-PS1 PrP^C^ KO, p>0.99; WT vs J20, p = 0.87; J20 vs J20 PrP^C^ KO, p>0.99). n = 5–8.

**Fig 8:**
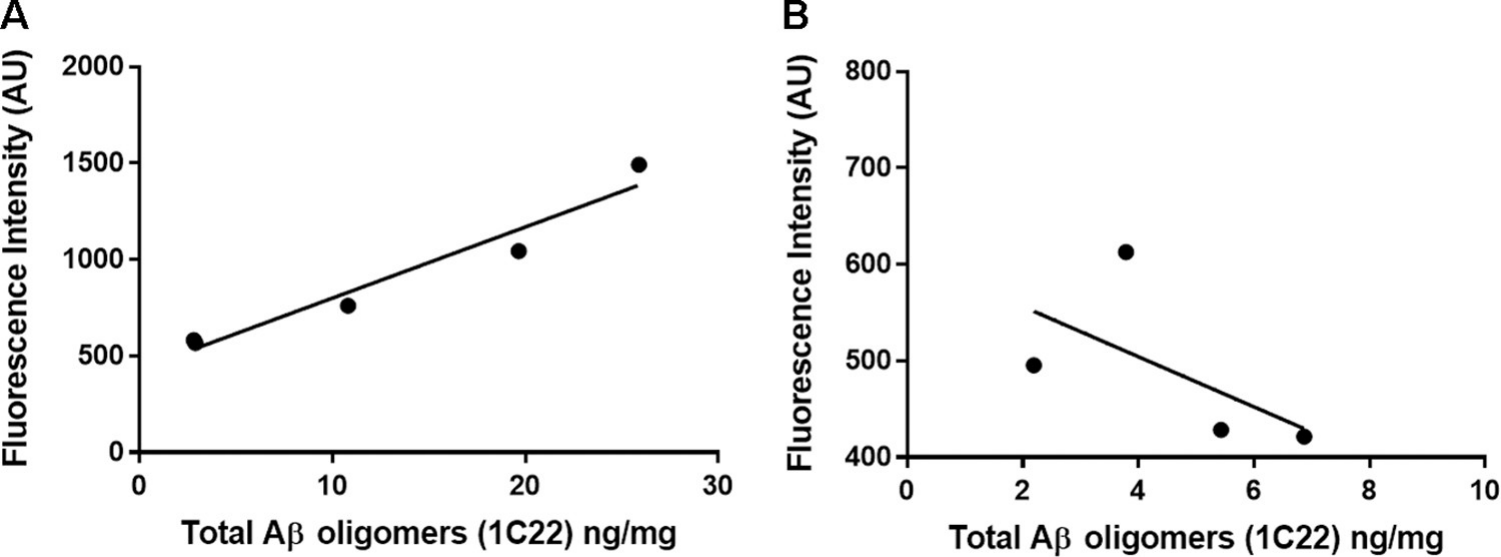
Positive correlation of Aβ oligomers that bind PrP^C^ with total amount of Aβ oligomers in APP-PS1, but not in J20 mice. **A)** Data from Figs 5 and 6 showed a positive correlation for APP-PS1 brain samples (Spearman r = 0.9, p = 0.04). n = 5. **B)** No correlation was found for values obtained using J20 brain samples (Spearman r = -0.8, p = 0.17). n = 4.

Similarly, when the levels of Aβ oligomers were quantified using the 1C22 immunoassay we found that APP-PS1 mice had significantly higher levels than their wild-type littermates, whereas there was no detectable significant difference between the J20 mice and their wild-type littermates, at 12 months of age (WT vs APP-PS1, p = 0.035; WT vs J20, p = 0.25). PrP^C^ expression had no impact on the levels of 1C22-reactive Aβ oligomers in either APP-PS1 or J20 transgenic lines (Fig 5). Analysis of the levels of Aβ oligomers capable of binding PrP^C^ revealed no significant differences between J20 mice and their respective wild type controls. In contrast, APP-PS1 samples contained significantly higher levels of these Aβ oligomers, with no differences detected between mice with *Prnp +/+* and *Prnp -/-* backgrounds (Fig 6). We then characterised the conformation of the Aβ oligomers, using the OC antibody which recognises parallel, in register fibrils (distinct from the A11 antibody, which binds to anti-parallel Aβ structures [25, 26]. A11 and OC antibodies recognise mutually exclusive epitopes and it has been suggested that A11 binds prefibrillar amyloid material, which could change conformation and aggregate into fibrils, while the OC fibrillar oligomers are protofibrils, transient intermediates, that ultimately become fibrils. These OC Aβ oligomers may represent fibril nuclei which are the minimal stable aggregate that is capable of elongating by recruiting additional monomers [25]. Interestingly, only APP-PS1 mice but not J20 mice, had a significant amount of these OC-Aβ oligomers (WT vs APP-PS1, p = 0.012; WT vs J20, p = 0.87) (Fig 7). It has been suggested that Aβ oligomers that are able to bind to PrP^C^ have an OC conformation [7, 27]. Levels of OC-Aβ oligomers did not change after ablation of PrP^C^ in either mouse line (Fig 7). Next, we hypothesised that the total level of Aβ oligomers in APP-PS1 would directly correlate to the amount of Aβ oligomers capable of binding PrP^c^. Interestingly, only APP-PS1 mice, and not J20 mice, showed a positive and significant correlation between total amount of Aβ oligomers and oligomers that bind to PrP^C^ (Fig 8). This is in agreement with previous studies which showed that J20 mice produce mainly A11-Aβ oligomers [28]. Given the low number of samples available in the current study, it would be helpful to further confirm this correlation with a bigger group size. Collectively, these results demonstrate that APP-PS1 mice produce more Aβ oligomers than J20 mice and that the oligomers from APP-PS1s are conformationally distinct and bind PrP^C^.

## Discussion

We used a range of biochemical and histological techniques to compared the impact of PrP^C^ expression on two different mouse models of AD, the APP-PS1 and J20 mouse lines. The ablation of PrP^C^ had no significant impact on the histological and biochemical end-points assessed. However, we found that the levels of Aβ plaques, peptides, oligomers, and PrP^C^-binding species differed between the two lines irrespective of PrP^C^ expression.

These observations provide a plausible explanation for the divergent results in which knock-out of PrP^C^ rescued the adverse phenotypes in APP-PS1 mice, but not in J20 mice (Cisse *et al*. and Gimbel *et al*). Specifically, our results suggest that PrP^C^-binding Aβ oligomers (which are abundant in APP-PS1, but not J20 mice) drive the phenotype seen in APP-PS1. Although performed with a low number of mice per group, our findings are consistent with a prior report which found that the proportion of Aβ oligomers that interact with PrP^C^ varies from model to model; however, that report did not investigate the J20 model [29]. These results highlight the need to characterise Aβ oligomers present in the AD mouse models and AD brain samples to understand the mechanisms of Aβ toxicity in AD.

In summary, our results provide a potential explanation for what appeared to be prior contradictory findings and highlight the need for thorough characterisation of Aβ oligomers, their binding to diverse receptors and subsequent effects on neurons. APP_swe_-PS1ΔE9 mice have proven to be an appropriate model to study Aβ-PrP^C^ interactions since they produce Aβ soluble fibrillary oligomers (OC-type) that bind PrP^C^. The existence of a ‘cloud’ of Aβ oligomer conformations poses the question whether their capability to trigger neurotoxicity depends on their structural conformation. Specific structures or strains associating with specific pathologies and clinical presentations is reminiscent of prion strains [30]. As there are many and diverse Aβ aggregates present in the brain, identifying the toxic Aβ species in AD is paramount for selection of models that recapitulate specific conformers of interest.

## Supporting information

S1 Raw images Western blot raw data for Fig 1. A) Whole Western blot images for APP and GADPH. B) Whole Western blot images for PrP^C^ and GADPH.
(PDF)
