# Supplementary material for: Two mouse models of Alzheimer’s disease accumulate amyloid at different rates and have distinct Aβ oligomer profiles unaltered by ablation of cellular prion protein
Source: PLoS One. 2023 Nov 17;18(11):e0294465. doi: 10.1371/journal.pone.0294465 (PMC10655998; doi:10.1371/journal.pone.0294465)
Supplement: S1 Raw images — A) Whole Western blot images for APP and GADPH. B) Whole Western blot images for PrPC and GADPH. (PDF) [file pone.0294465.s001.pdf]

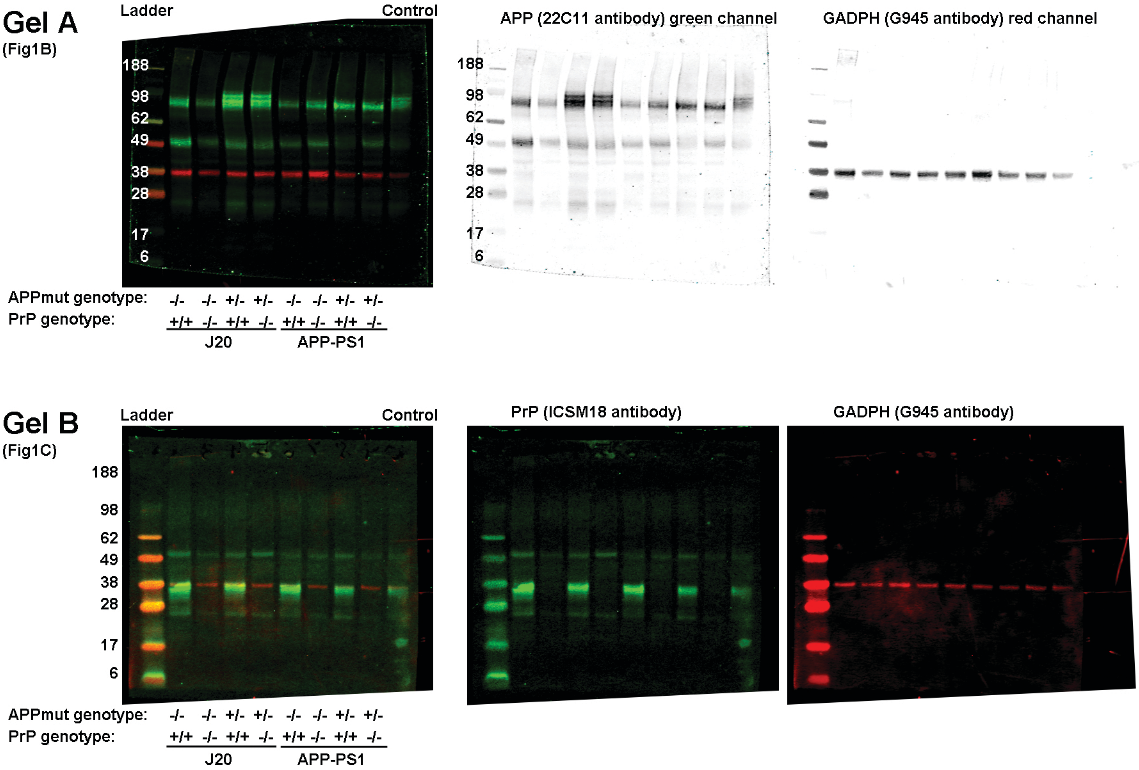
Gel A
(Fig1B)
Ladder
Control
188
98
62
49
38
28
17
6
APP (22C11 antibody) green channel
GADPH (G945 antibody) red channel
APPmut genotype: -/- -/- +/- +/- -/- -/- +/- +/-
PrP genotype: +/+ -/- +/+ -/- +/+ -/- +/+ -/-
J20
APP-PS1
Gel B
(Fig1C)
Ladder
Control
PrP (ICSM18 antibody)
GADPH (G945 antibody)
APPmut genotype: -/- -/- +/- +/- -/- -/- +/- +/-
PrP genotype: +/+ -/- +/+ -/- +/+ -/- +/+ -/-
J20
APP-PS1
